# Association between vaccination, viral antibodies, and asthma prevalence in the U.S.: insights from NHANES (1999–2020)

**DOI:** 10.3389/falgy.2025.1456934

**Published:** 2025-03-21

**Authors:** Zonghui Yang, Jia Guo, Manman Cheng, Youwen Zhang, Zhi Chen, Jie Wen, Fenglian Shan

**Affiliations:** ^1^ Clinical Medical College, Jining Medical University, Jining, China; ^2^ Department of Pulmonary and Critical Care Medicine, Affiliated Hospital of Jining Medical University, Jining, China

**Keywords:** NHANES, asthma, race, demographic factors, vaccine, viral antibody

## Abstract

**Objective:**

This investigation aimed to explore the differences in asthma prevalence among various demographic groups in the U.S., focusing on factors related to vaccination and viral antibodies.

**Methods:**

The study analyzed data from 37,445 individuals collected through the National Health and Nutrition Examination Survey between 1998 and 2020. Employing weighted sampling methods, the analysis considered the stratification and clustering typical of the survey's design. It particularly examined how age, race, income, smoke, education, and gender factors influence both the prevalence and severity of asthma.

**Results:**

This study aims to elucidate disparities in asthma prevalence across the U.S. population by examining the roles of demographic characteristics and factors related to vaccination and viral antibodies. It revealed a significant correlation between asthma prevalence and patient demographics, including age, gender, income, smoke, education, and race. We found that asthma patients were mostly found in participants with lower economic level (2.7 vs. 2.87). Non-Hispanic black women age exhibited a higher likelihood of asthma, at 17.7%, compared to non-Hispanic whites and Mexican Americans. Asthma prevalence peaks between the ages of 20 and 30 and has shown a rising trend over the years. Regarding vaccinations, hepatitis A, hepatitis B, pneumococcal, and HPV vaccines were associated with an increased risk of asthma. Conversely, patients testing positive for hepatitis A virus and core hepatitis B virus antibodies demonstrated a lower prevalence of asthma. Additionally, asthmatic patients showed lower average measles virus and rubella antibodies levels, at 0.53 and 3.32, respectively, compared to non-asthmatic individuals. Notably, asthma incidence was lower in herpesvirus I-positive patients (OR: 0.895, CI, 0.809%–0.991%), while herpesvirus II-positive patients displayed a higher incidence of asthma (OR: 1.102, CI, 0.974%–1.246%).

**Conclusion:**

The study findings underscore the significant prevalence of asthma and its correlation with population demographics, vaccination rates, and serum viral antibodies. These results highlight the importance of implementing tailored public health interventions.

## 1 Introduction

Asthma, a chronic lung disease, has experienced an increase in global prevalence over recent decades (1, 2). There are notable geographic and ethnic variations in the prevalence, severity, and mortality rates associated with asthma. High-income countries report higher asthma prevalence, yet most asthma-related deaths occur in low- and middle-income countries (3–5). The hygiene hypothesis posits that the increase in persistent asthma cases might be linked to enhancements in public health and hygiene (6), suggesting that early infections could confer protection by fostering the development of the T helper 1 (TH1) immunophenotype over the TH2 immunophenotype, which could elevate the risk of atopy (7, 8). Immunization might contribute to allergic diseases either by reducing exposure to infections that could otherwise offer natural immune system training or through direct immune-modulatory effects. The literature provides mixed evidence on the association between increased vaccination rates and the risk of atopic diseases such as asthma. Studies by Hurwitz, Morgenstern, and Yoneyama et al. support a correlation between DTP vaccination and increased atopic diseases (9, 10), while studies by Anderson and Wickens et al. report contrasting findings (11, 12). Vaccinations are pivotal in reducing morbidity and mortality from infectious diseases, necessitating further research in this field (13). Global health challenges such as viral infections including hepatitis A and B, herpes, and rubella have profound public health implications (14). These infections can exert systemic effects that extend beyond the initial site of infection, affecting various organ systems such as lymphatic, integumentary, renal, nervous, endocrine, cardiovascular, and respiratory systems (15–17). Several biological mechanisms have been hypothesized to explain how viral infections can influence lung health either directly or indirectly. Studies have linked viral infections to respiratory conditions such as interstitial lung disease, asthma, and COPD, underscoring the significant impact these viruses have on lung function (18–21). Despite the ongoing challenges in managing viral infections globally, the existing literature on the interplay between viral infections and respiratory conditions like COPD and asthma is still limited. While preliminary data suggest a potential link between vaccination, changes in viral antibodies, and the risk of asthma, further detailed research is essential. This study leverages individual-level data from the National Health and Nutrition Examination Survey (NHANES) to explore the possible association between viral antibodies and asthma risk, with an emphasis on ethnic variations.

## 2 Materials and methods

**Figure 1:**
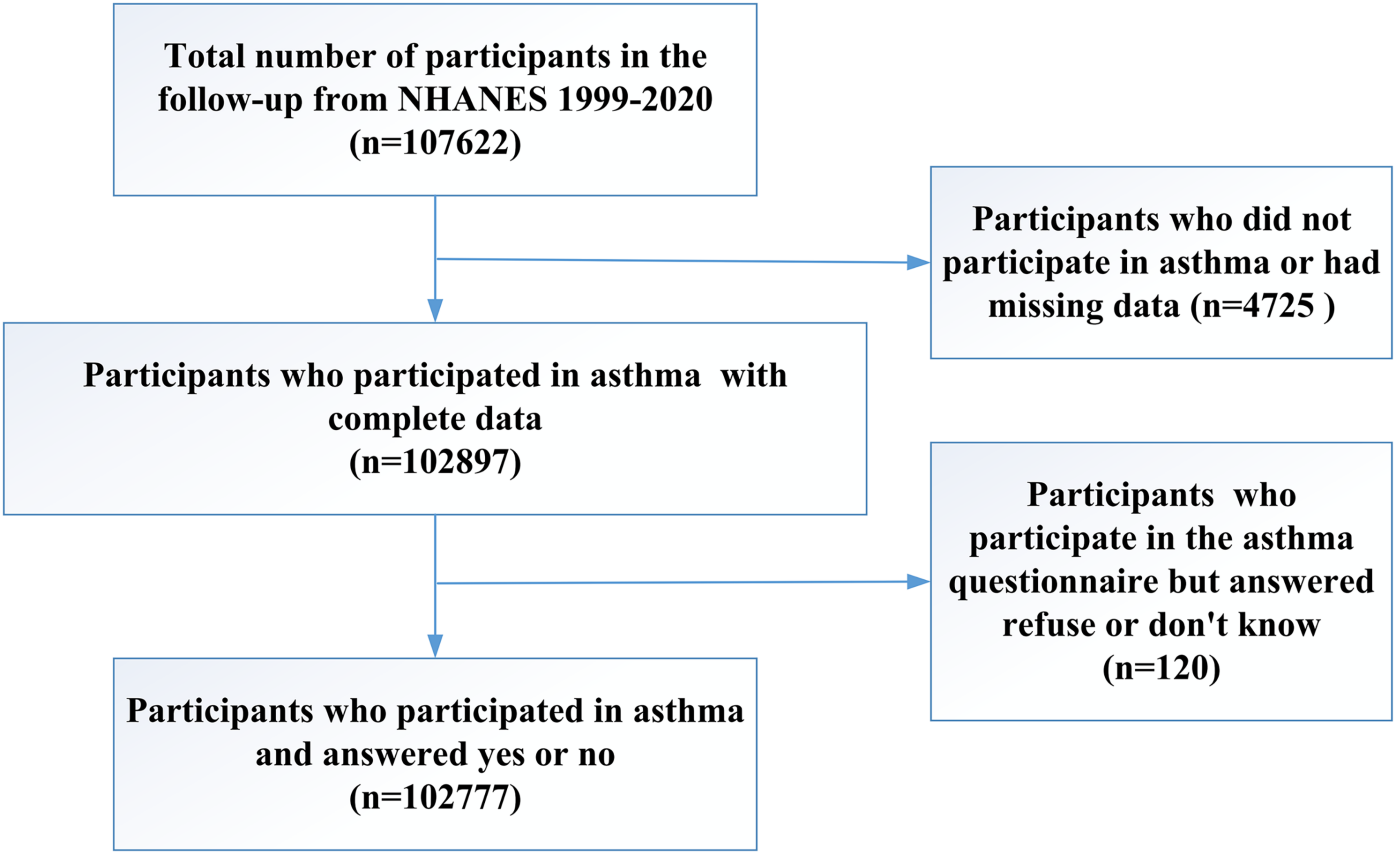
Flow diagram of participants inclusion for study.

This cross-sectional study utilized data from the National Health and Nutrition Examination Survey (NHANES) database, spanning from 1999–2020. NHANES is a collaborative initiative between the National Center for Health Statistics (NCHS) and the Centers for Disease Control and Prevention (CDC), designed to assess the nutritional and health status of the non-institutionalized U.S. population. NHANES employs a complex, multistage probability sampling design to collect nationally representative health-related data through interviews, mobile physical examinations, and laboratory tests. The program has been approved by the NCHS Research Ethics Review Board, and participation in the survey is contingent upon informed consent from all individuals involved. The data collection continued up to the onset of the COVID-19 pandemic in 2020. The 2019–2020 NHANES cycle was particularly impacted by the COVID-19 pandemic, leading to incomplete data collection that did not represent the national population adequately. Data from the 2017–2018 cycle were merged into the 2017–2020 NHANES cycle to create a comprehensive dataset for these years. Further details are accessible on the CDC website and other relevant sources [https://www.cdc.gov/nchs/nhanes/index.htm]. After excluding participants who lacked asthma information or did not participate in reproductive health questionnaires, the dataset included 102,777 participants (Figure 1).

The National Health and Nutrition Examination Survey (NHANES), approved by the Institutional Review Board (IRB) of the National Center for Health Statistics (NCHS), utilizes de-identified and publicly available data. Given this nature, ethical approval was not required. The study extracted data on vaccination status, serum viral antibody levels, and general clinical characteristics from patients with doctor-diagnosed asthma in NHANES, excluding any missing values. NHANES includes a section that captures individuals' self-reported health status, focusing on medical conditions (22). Asthma diagnosis confirmation relies on responses to two key questions: “Has a doctor or other health professional ever diagnosed you with asthma?” These queries were administered by trained interviewers at participants' homes using a Computer-Assisted Personal Interviewing (CAPI) system (23), which includes built-in validation checks to ensure data accuracy and consistency. The vaccination and serum virus antibody questionnaire covers hepatitis A virus, hepatitis B virus, pneumococcal, and HPV vaccines. The serum antibodies assessed include hepatitis A, hepatitis B surface, hepatitis B core, herpes virus, HPV, HPV, measles, and rubella antibodies.

Weighted samples were employed in all analyses to account for stratification and clustering, thus providing estimates representative of the entire U.S. population. To encompass a 22-year period, a variable weight sample was constructed by aggregating one-fifth of each individual's biennial weight from 1999–2020. RStudio, version 2022.07.0, running on a Windows platform (RStudio, PBC), in conjunction with R, version 4.2.1 (R Foundation for Statistical Computing), facilitated all statistical analyses. Initial frequency distributions and asthma prevalence were calculated across different age groups, genders, income and racial backgrounds without adjustment, following the standard age categorizations of detailed NHANES studies. Linear regression analyses comparing age-adjusted, gender-adjusted body measurements incorporated age as a continuous variable. Logistic regression models, adjusted for age, gender, education, income, smoke, and gender, assessed the impact of vaccination and serum virus antibody use on asthma. Although complex models that included interactions between gender and race, as well as age and race (treated as a continuous variable) across various age groups were explored, these models did not significantly enhance the model's predictive capability.

## 3 Results

**Figure 2:**
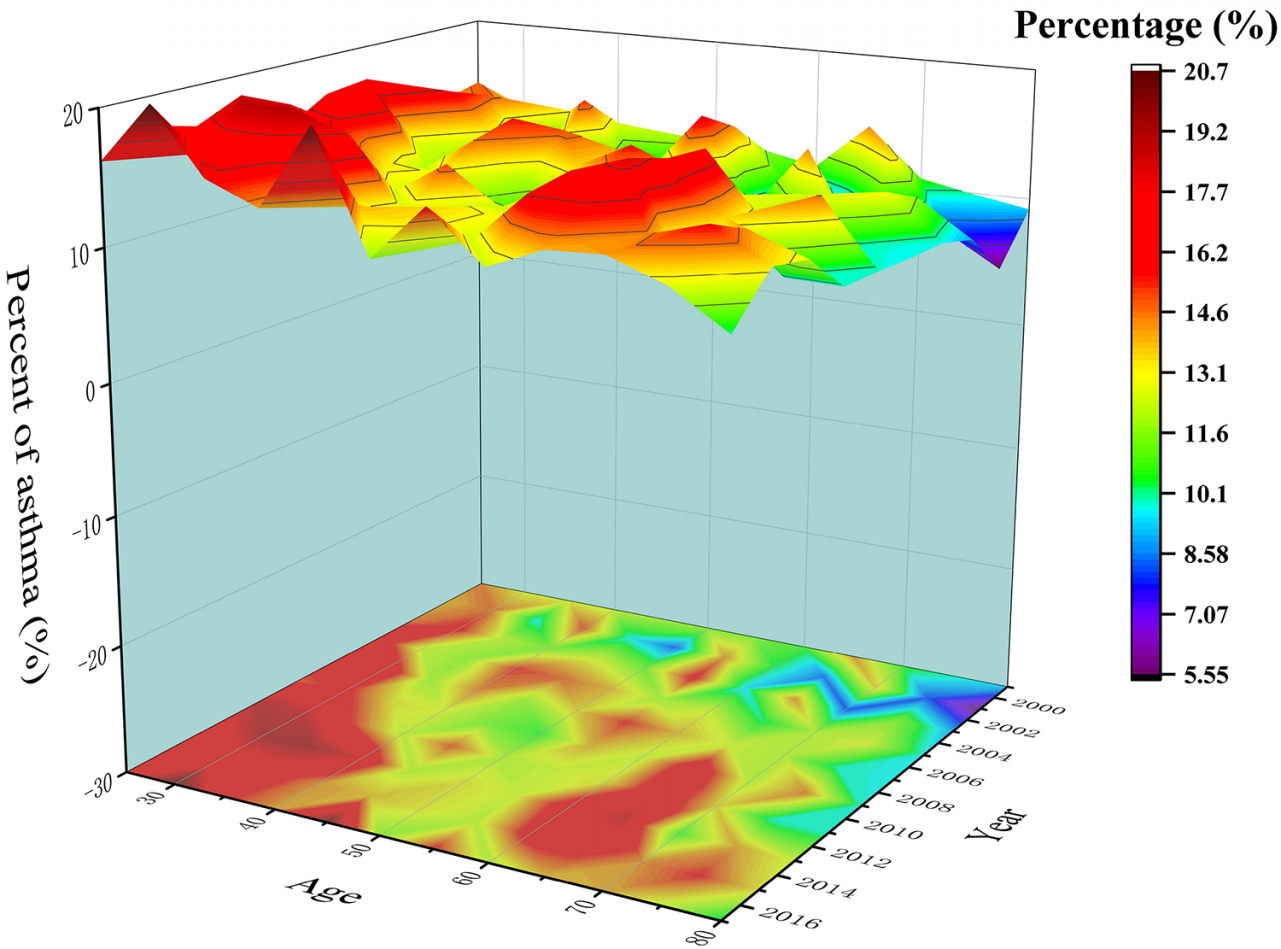
The trend of asthma incidence from 1999–2020 by age groups and year.

The study included a total of 37,445 participants from the NHANES dataset, representing approximately the U.S. residents. The analysis revealed a consistent and gradual increase in asthma prevalence over the course of the past decade. This trend was observed across different racial, gender, and age groups (Figure 2).

### 3.1 Demographic characteristics

**Figure 3:**
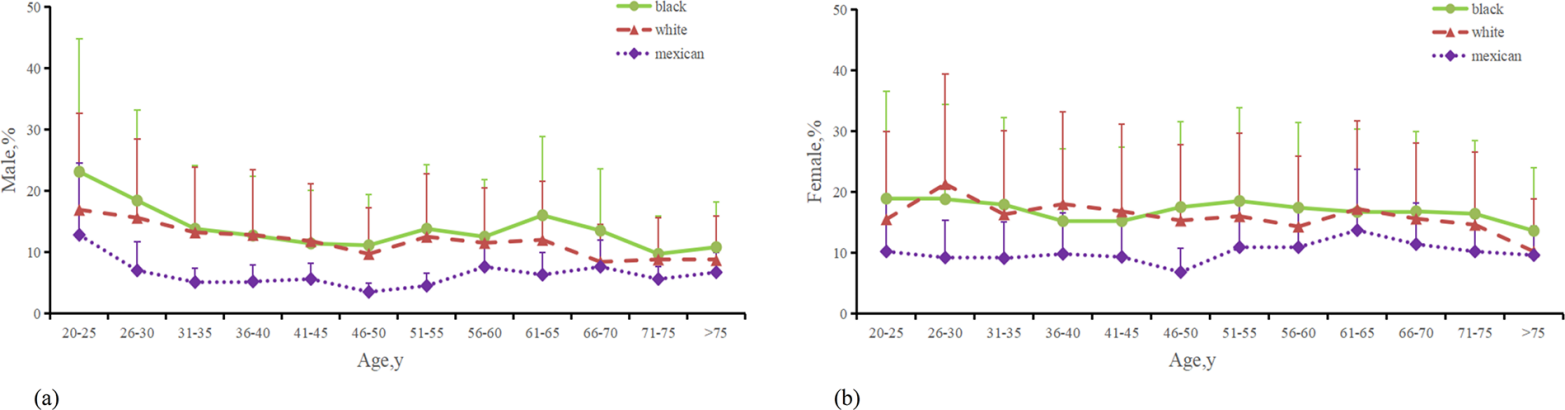
Distribution of the percentage of male and female with asthma. **(a)** Percentage of Males with asthma from 3 ethnic groups. **(b)** Percentage of Males with asthma from 3 ethnic groups. **(c)** Last menstrual period age in persons with asthma from 3 ethnic groups. (The Mexican in the figure represents Mexican Americans, the black represents non-Hispanic blacks, and the white represents non-Hispanic whites).

A total of 102,777 individuals participated in this study to determine the prevalence of asthma, of which 14,770 were diagnosed with asthma, representing 14.5% (CI, 14.1%–14.9%) of the total sample. The study highlighted variations in asthma prevalence across different demographic factors, such as age, gender, income, education, and race (Figure 3, Supplementary Appendix Tables S1, S15). Data spanning 22 years were utilized to estimate the asthma prevalence in the U.S. population. The analysis indicated that asthma prevalence was 17.7% (CI, 16.9%–18.4%) among Non-Hispanic black subjects, 14.6% (CI, 14.0%–15.1%) among Non-Hispanic white subjects, and 9.52% (CI, 8.86%–10.2%) among Mexican-American subjects. Among racial groups, Non-Hispanic black individuals exhibited a higher likelihood of having asthma, with Non-Hispanic black women showing a slightly higher prevalence than Non-Hispanic black men (17.7% vs. 17.6%). Conversely, Non-Hispanic white and Mexican-American individuals reported lower rates, with Mexican-American men having the lowest prevalence at 9.17%, followed by Mexican-American women at 9.9%. The prevalence rates among Non-Hispanic white men and women were 13.2% and 15.8%, respectively. From 1999–2000–2020, the prevalence of asthma increased from 13.1%–15.3%, with rising incidence rates among various ethnic groups. Notably, asthma incidence does not correlate with age, as most cases occur in individuals under 50 years old, with the highest rates observed in the 20–25 age group (16%, CI, 16%–17%) and the 26–30 age group (16%, CI, 15%–18%). Non-smokers made up the majority of the participants (50.06%), and in the comparison of economic income, it was found that asthma patients were mostly found in participants with lower economic level (2.7 vs. 2.87). In comparisons of educational attainment, those with higher levels of education were less likely to have asthma. Across all age, education, gender and economic income categories, Non-Hispanic black participants were more likely to have asthma compared to Non-Hispanic white and Mexican participants, with Non-Hispanic white participants more likely than Mexican participants (Supplementary Appendix Table S2). The prevalence rate of asthma among Non-Hispanic black individuals significantly exceeded that of other races (*P* < 0.001, OR: 2.036, CI, 1.858%–2.231%), with a notable interaction observed between race and gender and income (*P* < 0.001, OR: 2.098, CI, 1.909%–2.306%).

### 3.2 Vaccine

**Figure 4:**
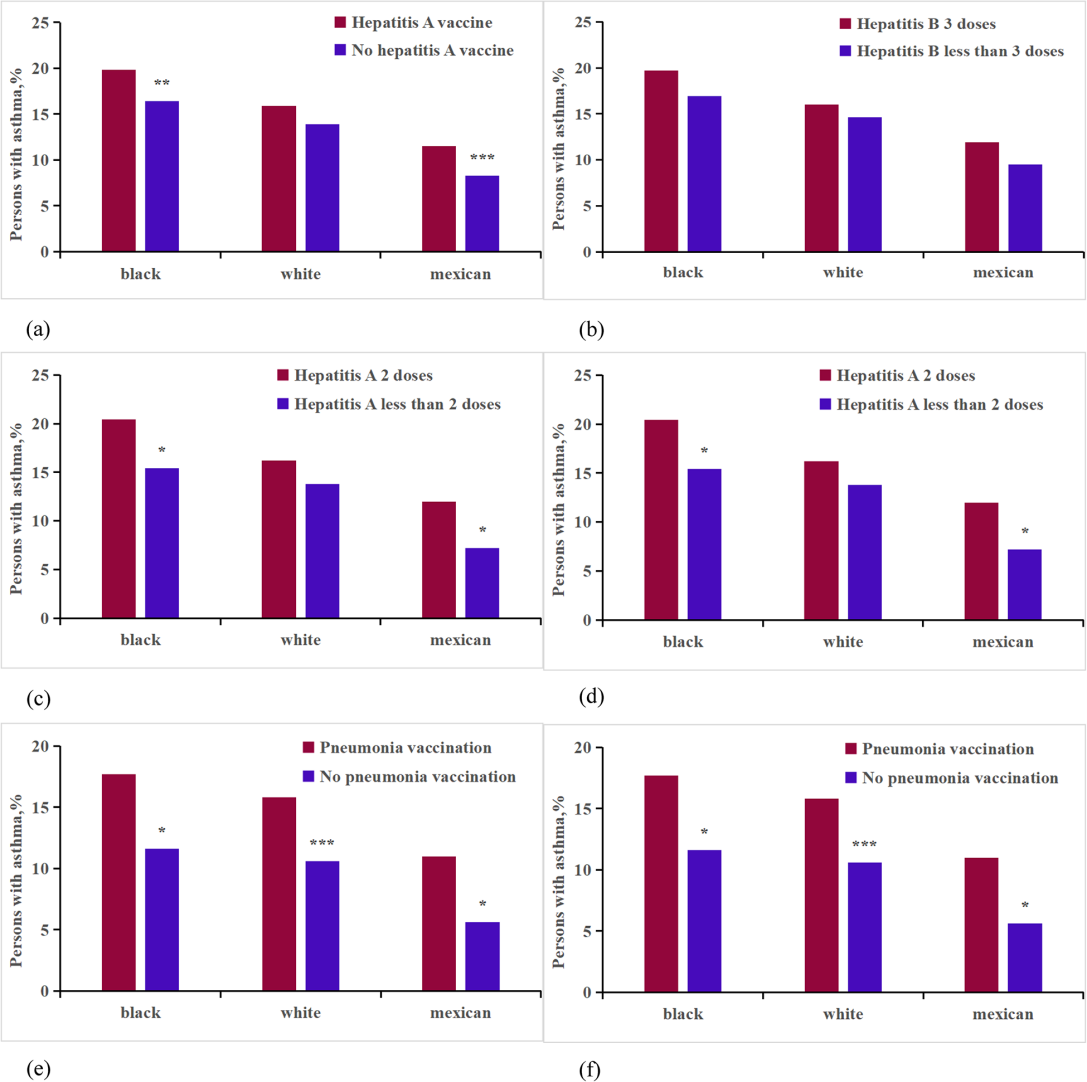
Analysis on the differences of vaccine factors between asthma patients and non-asthma participants of different races. ***p < 0.001; **p < 0.01;*p < 0.05. **(a)** Hepatitis A vaccine; **(b)** Hepatitis A vaccine dose;**(c)** Hepatitis B vaccine; **(d)** Hepatitis B vaccine dose; **(e)** Pneumonia vaccine; **(f)** HPV vaccine.

In our study, we compared the use of the hepatitis A virus vaccine, adjusted for age, race, income, smoke, education, and gender, between asthmatic and non-asthmatic individuals among vaccinated patients (Supplementary Appendix Table S3, Figure 4). For Non-Hispanic blacks, the number of vaccinated individuals was 7,757, compared to 14,106 unvaccinated; for Non-Hispanic whites, 8,733 vaccinated vs. 24,266 unvaccinated; and for Mexican Americans, 7,092 vaccinated against 10,935 unvaccinated. Our findings indicated a higher incidence of asthma among patients who received the hepatitis A virus vaccine compared to those unvaccinated. This difference was statistically significant (*P* < 0.001, OR: 1.178, CI, 1.111%–1.249%), with elevated rates observed particularly among Non-Hispanic white and Mexican participants. Notably, there was a substantial impact on growth (0.088%, 0.094%). Logistic regression analysis, adjusting for age, race, income, smoke, education, and gender, revealed that vaccination was associated with an increased risk of hepatitis A virus infection compared to non-vaccination (*P* < 0.01, OR: 1.132, CI, 1.041%–1.231%). Additionally, individuals who received two doses of the hepatitis A virus vaccine exhibited a higher incidence rate (*P* < 0.05, OR: 1.285, CI, 1.043%–1.583%) compared to those who received only one dose, particularly among Non-Hispanic black and Mexican populations (Supplementary Appendix Tables S4, S16, Figure 4).

We conducted a comparison of asthma rates between participants vaccinated against hepatitis B virus and those who were not (Supplementary Appendix Table S6, Figure 4). For Non-Hispanic blacks, the number of vaccinated individuals was 12,321 compared to 956 non-vaccinated individuals; for Non-Hispanic whites, the figures were 16,217 vaccinated vs. 18,531 non-vaccinated; and for Mexican Americans, 10,937 were vaccinated compared to 7,970 non-vaccinated. After analyzing the data for heterogeneity, we found a statistically significant difference in the overall average percentage of asthma incidence between the vaccinated and non-vaccinated groups (*P* < 0.001, OR: 1.145, CI, 1.063%–1.233%). However, no significant relationship was found between asthma incidence and the dosage of the hepatitis B virus (*P* > 0.05, OR: 1.193, CI, 0.970%–1.468%) (Supplementary Appendix Tables S5, S16, Figure 4).

The study involved 14,954 participants who were surveyed regarding pneumococcal vaccination status (Supplementary Appendix Tables S7, S16, Figure 4). Among these participants, 373 Non-Hispanic black individuals had received the vaccine, while 2,439 had not; 2,148 Non-Hispanic white individuals were vaccinated, compared to 5,444 who were not; and 345 Mexican individuals had received the vaccine, with 2,982 remaining unvaccinated. After adjusting for age, race, income, smoke, education, and gender, the analysis revealed that individuals who received the pneumococcal vaccine exhibited a 0.78% higher incidence of asthma compared to those who did not receive the vaccine (*P* < 0.001, OR: 2.132, CI, 1.656%–2.744%). This increase was more pronounced in Non-Hispanic white individuals, with an incidence increase of 0.94%, compared to 0.62% in Non-Hispanic blacks, and 0.82% in Mexicans.

In our final analysis, we assessed the presence of HPV vaccination within the dataset (Supplementary Appendix Tables S8 and S16, Figure 4). It was found that 1,268 Non-Hispanic blacks, 1,462 Non-Hispanic whites, and 815 Mexicans had received the HPV vaccine. In contrast, 6,011 Non-Hispanic blacks, 8,576 Non-Hispanic whites, and 4,230 Mexicans had not been vaccinated against HPV. Significant differences in asthma prevalence were observed between those vaccinated for HPV and those who were not (*P* < 0.001, OR: 1.577, CI, 1.418%–1.755%). The analysis stratified by race indicated that HPV vaccination significantly impacted the increase in asthma rates among Non-Hispanic white, Non-Hispanic black, and Mexican participants, with increases ranging from 0.28%–0.29%. After adjusting for confounding factors, the average incidence of receiving the HPV vaccine was higher among vaccinated respondents compared to unvaccinated individuals (*P* < 0.001, OR: 1.329, CI, 1.346%–1.600%). However, no significant relationship was found between asthma incidence and the dosage of the HPV vaccine (*P* > 0.05, OR: 0.916, CI, 0.648%–1.278%).

### 3.3 Virus serum antibodies

**Figure 5:**
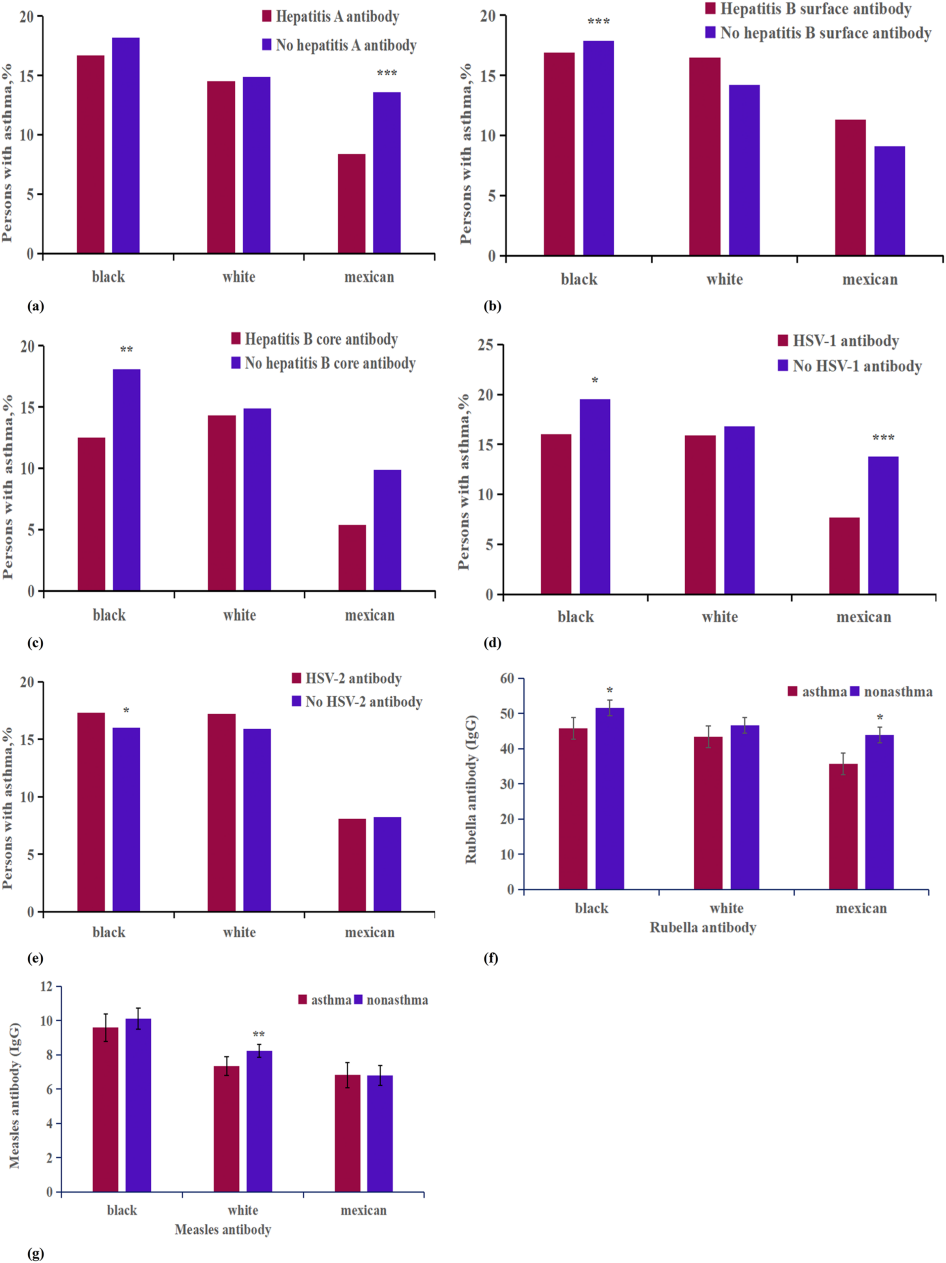
Analysis on the differences of virus antibody factors between asthma patients and non-asthma participants of different races. ***p < 0.001; **p < 0.01; *p < 0.05. **(a)** Hepatitis A antibody; **(b)** Hepatitis B surface antibody; **(c)** Hepatitis B core antibody; **(d)** HSV-1 antibody; **(e)** HSV-2 antibody; **(f)** Measles antibody; **(g)** Rubella antibody.

In our study of patients with viral antibodies, we compared populations with hepatitis A antibodies, adjusted for age, race, income, smoke, education, and gender, between asthmatic and non-asthmatic participants (Supplementary Appendix Tables S9, S16, Figure 5). The data showed that the numbers of hepatitis A virus positive and negative individuals were 8,383 and 11,082 for Non-Hispanic blacks, 10,430 and 20,998 for Non-Hispanic whites, and 13,301 and 4,504 for Mexican Americans, respectively. Our analysis indicated a significant association between hepatitis A antibody negativity and an increased percentage of asthma cases among participants of Mexican descent, with a 0.51% increase observed in the racial breakdown. Additionally, in a separate logistic regression analysis that controlled for age group, race, and hepatitis A virus vaccination status, we found that individuals negative for hepatitis A virus antibodies had a higher incidence rate of asthma compared to those who were antibody positive (*P* < 0.001, OR: 0.864, CI, 0.797%–0.937%).

Next, we assessed the prevalence of asthma among individuals with positive and negative tests for hepatitis B virus antibodies (Supplementary Appendix Table S10, S11, S16, Figure 5). For hepatitis B surface antibodies, among Non-Hispanic blacks, 7,380 tested positive and 12,074 tested negative; among Non-Hispanic whites, 8,198 tested positive and 23,277 tested negative; and among Mexican Americans, 5,844 tested positive and 11,853 tested negative. For hepatitis B core antibodies, the numbers were 1,535 positive and 16,490 negative for Non-Hispanic blacks, 801 positive and 29,085 negative for Non-Hispanic whites, and 309 positive and 15,991 negative for Mexican Americans. A heterogeneity analysis revealed that among Non-Hispanic blacks, individuals with positive tests for both hepatitis B surface and core antibodies exhibited a lower incidence of asthma compared to those testing negative. In the adjusted analysis, only the presence of core antibodies was associated with a reduced incidence of asthma (*P* < 0.05, OR: 0.866, CI, 0.749%–1.001%); surface antibodies did not show a statistically significant difference (*P* > 0.05, OR: 0.802, CI, 0.443%–1.451%).

The questionnaire data on herpes virus exposure showed distinct patterns among participants (Supplementary Appendix Table S16, Figure 5). For herpes virus 1, there were 4,468 Non-Hispanic black individuals who tested positive and 3,126 who tested negative; 5,527 Non-Hispanic white individuals tested positive while 6,488 tested negative; and 5,709 Mexican Americans tested positive compared to 2,099 who tested negative. For herpes virus 2, there were 2,175 Non-Hispanic black individuals testing positive and 3,401 testing negative; 1,371 Non-Hispanic white individuals tested positive, against 8,750 testing negative; and 664 Mexican Americans tested positive while 5,031 tested negative. Analysis indicated that herpes virus 1-positive patients exhibited a lower incidence of asthma (*P* < 0.05, OR: 0.895, CI, 0.809%–0.991%), whereas herpes virus 2-positive patients had a higher incidence of asthma (*P* < 0.05, OR: 1.102, CI, 0.974%–1.246%). Racial analysis further revealed that the impact of herpes virus 1 positivity on asthma was more significant among Non-Hispanic black and Mexican populations, while herpes virus 2 positivity had a greater impact only among Non-Hispanic black individuals (Supplementary Appendix Tables S12, S13).

**Figure 6:**
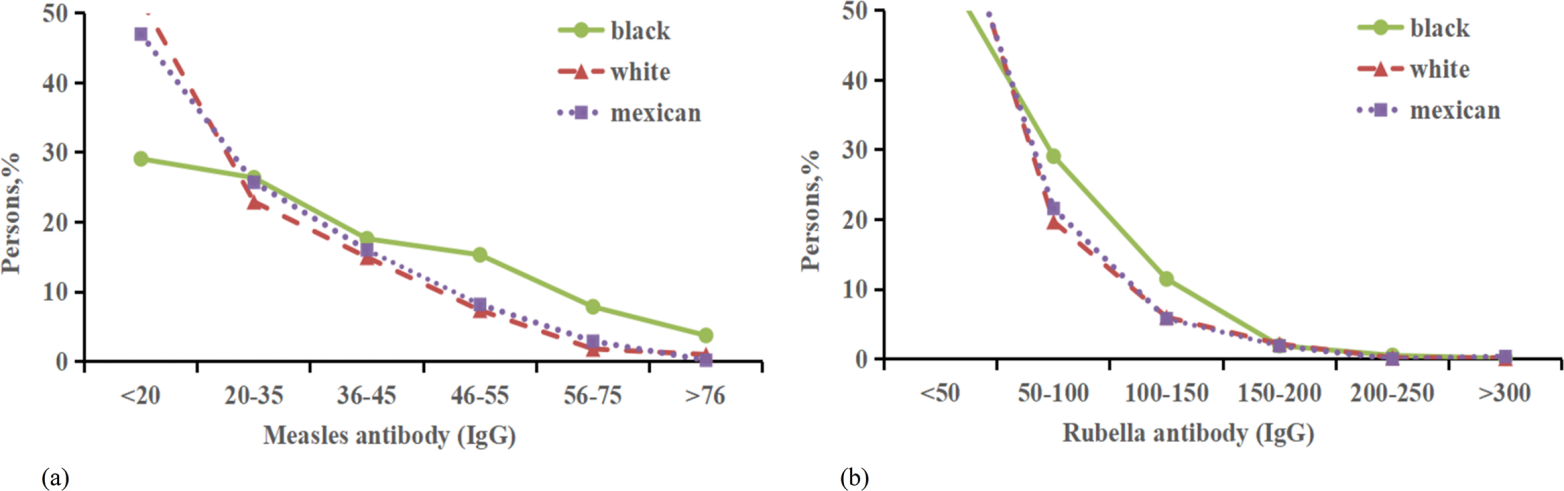
Distribution of the percentage of measles antibody and Rubella antibody with asthma. **(a)** Percentage of Rubella antibody (IgG) negative with asthma from 3 ethnic groups. **(b)** Percentage of Rubella antibody (IgG) negative with asthma negative from 3 ethnic groups.

In this study, measles, rubella, and varicella virus antibodies, adjusted for age, gender,race,incme,smoke and education were compared between asthmatic and non-asthmatic patients (Supplementary Appendix Tables S14, S16, Figure 5). The sample included 1,125 Non-Hispanic black participants with asthma and 4,795 without; 1,220 Non-Hispanic white participants with asthma and 6,900 without; and 643 Mexican American participants with asthma and 6,080 without. On average, measles virus antibodies and rubella antibodies were found to be lower in asthmatic patients compared to their non-asthmatic counterparts, with average levels of 0.53 and 3.32, respectively (Figure 6). Moreover, asthma was associated with a more pronounced decrease in varicella antibodies among Mexican subjects (7.67) than among Non-Hispanic white subjects (1.94).

## 4 Discussion

### 4.1 Demographic disparities in asthma prevalence

Demographic characteristics significantly influence the prevalence of asthma, with factors varying based on race, nationality, and social determinants such as cultural backgrounds, ethnicities, and living areas (24). Data from the Centers for Disease Control and Prevention reveal that in the United States, certain racial and ethnic groups, such as Black individuals, have a higher likelihood of asthma diagnosis compared to White and Mexican Americans, which aligns with our findings (25–27). During the COVID-19 pandemic, White, Black, and Latino populations face an increased risk for asthma due to factors including viral infections, income, education, occupation, housing, food security, and social support (28, 29). Our analysis addresses previous data limitations and extends the understanding that asthma prevalence varies by gender and age. Specifically, our study shows a higher incidence of asthma among young women, particularly in the 20–30 age group, consistent with international research (30). These age-related differences in asthma prevalence are consistent across different ethnic groups. Age emerges as a key determinant in asthma incidence. Our analysis highlights that young non-Hispanic Black women have the highest prevalence of asthma, corroborating similar findings in other studies. And by comparing participants' incomes, we found that asthma prevalence was higher among participants with lower incomes and education. In the study of smokers, it is noteworthy that our findings indicated a predominance of non-smokers among both asthmatic and non-asthmatic participants. International research has established that smoking is a significant risk factor for the development of prevalent respiratory diseases, including lung cancer, chronic obstructive pulmonary disease (COPD), and bronchial asthma. Furthermore, smoking may influence the recruitment and activation of various immune cells, such as macrophages, dendritic cells, mast cells, natural killer cells, T lymphocytes, and B lymphocytes in patients (31). This observation may be correlated with the substantial proportion of non-smokers within our participant group. However, the underlying reasons for these differences warrant further investigation. This population-based analysis underscores the significant genetic influences on asthma and reinforces the consistent and robust differences observed across age, race, education, income, and gender (32–35). This may be the result of socio-economic differences between racial groups, as race is a complex structure of genetic, environmental and social factors, and historical practices such as redlining in the United States have categorized communities with high concentrations of blacks and other racial minorities as less economically desirable, and blacks and Hispanics are likely to use fewer health care services and medications compared to white (36, 37). And individuals with higher educational attainment are likely to manage their asthma medications more effectively, resulting in superior asthma control compared to their less educated counterparts. Conversely, those with lower levels of education may struggle to achieve optimal asthma control. However, based on the current analyses, we are unable to definitively ascertain which specific aspects of educational attainment are most critical in their relationship with asthma control.

### 4.2 Association and potential biological mechanisms between inoculation, viral antibodies and asthma

In a detailed analysis of individual-level data from the US population, researchers identified a correlation between widespread vaccination and an increased risk of asthma, potentially linked to immune system alterations. Specifically, individuals vaccinated against hepatitis A, hepatitis B, pneumococcal disease, and HPV showed a higher likelihood of developing asthma (38, 39). This association may be due to disruptions in the balance of serum antibodies in individuals with asthma, resulting in local and systemic eosinophilia, elevated levels of total and allergen-specific serum IgE and IgG4 antibodies, and Th2-mediated lung inflammation. The effectiveness of vaccinations in the asthmatic population continues to be debated. A study by Marshall et al. indicated that patients with atopic asthma had higher secretion levels of IL4 and IL5 post-vaccination, cytokines critically involved in asthma pathogenesis (40). Vaccination may potentially stimulates IL-1 cytokine production in human immune cells (41), which in turn triggers the release of various proinflammatory cytokines such as IL-6 and enhances antigen-driven responses by CD4 and CD8T cells. This heightened inflammatory response in sensitized individuals may elevate the risk of developing asthma (42, 43).

Our research indicated a lower prevalence of asthma among individuals who tested positive for serum hepatitis A virus and core hepatitis B virus antibodies, consistent with several international studies. A study in Turkey showed that children with hepatitis A IgG antibodies had a reduced risk of atopy, specific IgE positivity, and allergic conditions such as allergic rhinitis and asthma. This association appeared influenced by factors including age, paternal education level, and the presence of older siblings, rather than mere quantity (44). The underlying mechanism may be related to unique gene variants of TIM-1, the human cell surface receptor for HAV, which could inhibit Th2 differentiation through binding to TIM-1 (45, 46). Regarding hepatitis B infection, CD8T cells producing interferon gamma and tumor necrosis factor (TNF)-α may influence regulatory T lymphocytes, altering the Th1/Th2 balance. Research on herpesviruses has shown contrasting results; herpesvirus I-positive patients exhibited a lower incidence of asthma, while herpesvirus II-positive patients displayed a higher prevalence of asthma. This discrepancy could be linked to the effects of infection on cytokine balance, but it still requires further investigation. While limited global research has addressed the relationship between HSV and allergic diseases, existing studies offer conflicting outcomes. Some researchers suggest that HSV may protect against allergic conditions, whereas others propose it exacerbates them (47, 48). Various studies have presented differing views on the relationship between HSV and atopy, but they collectively affirm an association between HSV-1 infection and atopy, aligning with our findings. A retrospective study suggested a potential negative impact of asthma on the rapid decline of measles antibodies. However, there is minimal research on the correlation between rubella virus antibodies and asthma (49). Our findings show that mean levels of measles and rubella virus antibodies are lower in asthmatic patients compared to non-asthmatic individuals. The underlying mechanisms remain unclear and could involve factors such as variations in plasma cell lifespan and humoral immunity duration. In summary, there may be a plausible link between asthma, vaccination, and viral antibody levels, representing a potentially significant but overlooked immunological aspect of asthma. Further research is needed to explore this potential association more deeply.

### 4.3 Study limitations and future research directions

Asthma is a heterogeneous disease influenced by a combination of immune, genetic, and environmental factors, involving various cell types. It is closely related to environmental conditions, lifestyle choices, dietary patterns, and numerous other factors. International studies have demonstrated that living near highways, industrial areas, or rivers, sharing bedrooms, cooking with gas, having furry pets, and exposure to airborne pollutants may potentially trigger or exacerbate asthma (50, 51). Residents in areas with high levels of environmental pollution may experience increased health issues, leading to strained healthcare resources and insufficient health awareness, which may, in turn, affect vaccination rates. Additionally, unhealthy diets, characterized by high sugar and high fat, can result in a reduced intake of antioxidants, thereby increasing the risk of respiratory inflammation (52). These confounding factors may interact with one another, further heightening the risk of asthma. But this study was based on cross-sectional data from NHANES, it was not possible to fully incorporate these confounding factors and given the possible time-lagged effects between vaccination and asthma development and data limitations, causality was not established, but rather potential associations that require further prospective study were identified. Future prospective cohort studies should assess these factors and their interrelationships in more detail.

Our research has several strengths. Firstly, it encompasses 22 years of national survey data from 1999–2020, providing a substantial dataset. Secondly, our study offers temporal continuity over a long duration, a feature lacking in many international studies. Additionally, we focused on analyzing the relationship between asthma and vaccine and viral antibodies across different ethnic groups, a topic with limited international research, which mostly centers on non-Hispanic Blacks and non-Hispanic Whites. However, our study has limitations. Firstly, we concentrated on asthma prevalence and did not delve into associated complications. Secondly, we examined asthma data as a whole without disaggregating between specific asthma types. Moreover, limitations in racial information collection in the NHANES database led us to focus solely on adult women from three major American racial groups, potentially excluding valuable insights from other racial groups such as Asian Americans. Additionally, our data is derived from a public database, the impact of the COVID-19 pandemic on NHANES data collection from 2019–2020 may have introduced potential biases due to incomplete data collection. To address these limitations, a well-designed prospective cohort trial is necessary to validate our findings.

## 5 Conclusion

In this cross-sectional study, significant and consistent associations were identified between asthma occurrence and age, gender, income, smoke, education, and race among adult in the United States from 1999–2020. Factors such as vaccination and serum virus antibody were found to potentially increase the risk of asthma. Further research is needed to fully understand the causal relationship between vaccination and serum virus antibody factors and asthma in American adult.

## Data Availability

Publicly available datasets were analyzed in this study. This data can be found here: https://www.cdc.gov/nchs/nhanes/index.htm.
