# Supplementary material for: Association between vaccination, viral antibodies, and asthma prevalence in the U.S.: insights from NHANES (1999–2020)
Source: Front Allergy. 2025 Mar 21;6:1456934. doi: 10.3389/falgy.2025.1456934 (PMC11968725; doi:10.3389/falgy.2025.1456934)
Supplement: Supplementary file 1 [file Table1.docx]

**Appendix Table 1. Percentage of Participants with asthma**

| Sex and Age Group | Participants, n(%) [95% CI] | | |
| --- | --- | --- | --- |
|  | Black | White | Mexican-American |
| **Females** |  |  |  |
| 20-25 y | 1172(18.9)[17.7-20.1] | 998(15.5)[14.4-16.7] | 589(10.2)[9.1-11.2] |
| 26-30 y | 113(18.8)[15.6-22.1] | 210(21.3)[18.1-24.4] | 42(9.2)[6.2-12.2] |
| 31-35 y | 102(17.9)[14.3-21.4] | 164(16.3)[13.7-18.8] | 37(9.1)[5.9-12.3] |
| 36-40 y | 82(15.2)[11.9-18.6] | 183(18)[15.2-20.8] | 46(9.8)[6.8-12.8] |
| 41-45 y | 97(15.2)[12.2-18.3] | 157(16.8)[14.3-19.3] | 45(9.3)[6.3-12.2] |
| 46-50 y | 94(17.5)[14-21] | 161(15.3)[12.5-18] | 25(6.8)[4-9.6] |
| 51-55 y | 103(18.5)[15.3-21.7] | 168(16)[13.6-18.5] | 38(10.9)[7.4-14.3] |
| 56-60 y | 91(17.4)[14-20.8] | 136(14.3)[11.6-17] | 39(10.9)[6.4-15.3] |
| 61-65 y | 108(16.7)[13.6-19.7] | 157(17.2)[14.5-19.8] | 56(13.7)[10-17.5] |
| 66-70 y | 74(16.8)[13.1-20.6] | 129(15.6)[12.4-18.8] | 31(11.4)[6.8-16.1] |
| 71-75 y | 46(16.4)[12-20.9] | 118(14.6)[11.9-17.4] | 18(10.2)[6-14.5] |
| >75 y | 62(13.6)[10.4-16.8] | 200(10.2)[8.6-11.8] | 25(9.6)[4.8-14.3] |
| **Male** |  |  |  |
| 20-25 y | 1495(23.1)[21.7-24.5] | 1154(16.9)[15.7-18.1] | 785(12.8)[11.7-13.9] |
| 26-30 y | 85(18.4)[14.8-21.9] | 119(15.6)[12.8-18.4] | 33(7)[4.7-9.3] |
| 31-35 y | 66(13.8)[10.3-17.2] | 130(13.2)[10.6-15.8] | 17(5.1)[2.3-8] |
| 36-40 y | 60(12.7)[9.7-15.6] | 132(12.8)[10.7-14.8] | 19(5.2)[2.7-7.6] |
| 41-45 y | 59(11.4)[8.7-14.2] | 102(11.8)[9.4-14.3] | 21(5.6)[2.6-8.6] |
| 46-50 y | 57(11.1)[8.3-14] | 99(9.7)[7.5-11.9] | 13(3.5)[1.4-5.7] |
| 51-55 y | 69(13.8)[10.4-17.2] | 137(12.5)[10.3-14.8] | 15(4.5)[2.1-6.9] |
| 56-60 y | 57(12.5)[9.3-15.8] | 120(11.5)[9-14.1] | 23(7.6)[4.5-10.7] |
| 61-65 y | 98(16)[12.8-19.3] | 101(12)[9.6-14.4] | 23(6.3)[3.6-8.9] |
| 66-70 y | 59(13.5)[10.1-16.9] | 73(8.4)[6.1-10.6] | 24(7.6)[4.4-10.8] |
| 71-75 y | 29(9.7)[6.2-13.2] | 84(8.8)[6.8-10.8] | 13(5.6)[2.1-9] |
| >75 y | 37(10.8)[7.4-14.3] | 169(8.7)[7.2-10.2] | 14(6.7)[3.2-10.1] |

**Appendix Table 2. Prevalence of asthma**

| Variable | Prevalence, %(95% CI) |
| --- | --- |
| **Ethnicity** |  |
| White | 17.7(16.9-18.4) |
| Black | 14.6(14-15.1) |
| Mexican American | 9.5(8.9-10.2) |
| **Sex** |  |
| Female | 15.5(14.9-16) |
| Male | 13.4(13-13.9) |
| **Age** |  |
| 20-25 y | 0.16(0.16-0.17) |
| 26-30 y | 0.16(0.15-0.18) |
| 31-35 y | 0.14(0.13-0.15) |
| 36-40 y | 0.14(0.13-0.15) |
| 41-45 y | 0.13(0.12-0.15) |
| 46-50 y | 0.12(0.11-0.13) |
| 51-55 y | 0.14(0.13-0.15) |
| 56-60 y | 0.13(0.12-0.15) |
| 61-65 y | 0.15(0.13-0.16) |
| 66-70 y | 0.12(0.11-0.14) |
| 71-75 y | 0.12(0.11-0.14) |
| >75 y | 0.1(0.09-0.11) |

**Appendix Table 3. Comparative Mean percentage of participants with asthma in hepatitis A vaccine**

| **Variable** | **Hepatitis A vaccine** | **No hepatitis A vaccine** | **Mean Difference between asthma and non asthma** | **P Value** |
| --- | --- | --- | --- | --- |
| Black | 19.8(18.5-21.2) | 16.4(15.6-17.1) | 0.13(0.03-0.24) | 0.009 |
| White | 15.9(14.8-16.9) | 13.9(13.4-14.5) | 0.09( -0.02-0.19) | 0.057 |
| Mexican-American | 11.5(10.5-12.6) | 8.3(7.5-9) | 0.26(0.15-0.37) | <0.001 |

**Appendix Table 4. Comparative Mean percentage of participants with asthma in hepatitis A vaccine dose**

| **Variable** | **2 doses** | **1 dose** | **Mean Difference between asthma and non asthma** | **P Value** |
| --- | --- | --- | --- | --- |
| Black | 20.4(18.9-21.8) | 15.4(12.6-18.2) | 0.27 (0.17-0.38) | 0.027 |
| White | 16.2(15.1-17.4) | 13.8(11.7-15.9) | 0.17(0.07-0.28) | 0.080 |
| Mexican-American | 12(10.8-13.1) | 7.2(4.7-9.8) | 0.52(0.41-0.62) | 0.015 |

**Appendix Table 5. Comparative Mean percentage of participants with asthma in hepatitis B vaccine**

| **Variable** | **Hepatitis B vaccine** | **No hepatitis B vaccine** | **Mean Difference between asthma and non asthma** | **P Value** |
| --- | --- | --- | --- | --- |
| Black | 19.5(18.5-20.5) | 15.4(14.5-16.3) | 0.14(0.04-0.25) | 0.011 |
| White | 15.9(15.1-16.7) | 13.3(12.7-13.9) | 0.11(0.005-0.22) | 0.006 |
| Mexican-American | 11.7(10.8-12.6) | 7.3(6.5-8.1) | 0.52(0.41-0.63) | <0.001 |

**Appendix Table 6. Comparative Mean percentage of participants with asthma in hepatitis B vaccine dose**

| **Variable** | **3 doses** | **Less than 3 doses** | **Mean Difference between asthma and non asthma** | **P Value** |
| --- | --- | --- | --- | --- |
| Black | 19.7(18.7-20.7) | 16.9(13.5-20.3) | 0.17(0.06-0.27) | 0.21 |
| White | 16(15.2-16.8) | 14.6(12.2-17.1) | 0.11 (0.005-0.22) | 0.27 |
| Mexican-American | 11.9(11-12.9) | 9.5(6.9-12.1) | 0.27(0.16-0.37) | 0.10 |

**Appendix Table 7. Comparative Mean percentage of participants with asthma in pneumonia vaccine**

| **Variable** | **pneumonia vaccine** | **No pneumonia vaccine** | **Mean Difference between asthma and non asthma** | **P Value** |
| --- | --- | --- | --- | --- |
| Black | 17.7(12.4-23.1) | 11.6(9.6-13.5) | 0.62(0.52-0.73) | 0.011 |
| White | 15.8(12.9-18.8) | 10.6(8.8-12.4) | 0.94(0.84-1.05) | <0.001 |
| Mexican-American | 11(5.7-16.3) | 5.6(4.1-7.1) | 0.82(0.72-0.93) | 0.022 |

**Appendix Table 8. Comparative Mean percentage of participants with asthma in HIV vaccine**

| **Variable** | **HIV vaccine** | **No HIV vaccine** | **Mean Difference between asthma and non asthma** | **P Value** |
| --- | --- | --- | --- | --- |
| Black | 25.3(22.6-28) | 18.3(17-19.6) | 0.28(0.17-0.39) | 0.001 |
| White | 23.5(20.9-26.2) | 16.5(15.4-17.6) | 0.28(0.18-0.39) | <0.001 |
| Mexican-American | 16.7(14.1-19.3) | 10.1(8.8-11.4) | 0.29(0.18-0.39) | 0.032 |

**Appendix Table 9. Comparative Mean percentage of participants with asthma in hepatitis A antibody (Anti-HAV)**

| **Variable** | **Anti-HAV positive** | **Anti-HAV negative** | **Mean Difference between asthma and non asthma** | **P Value** |
| --- | --- | --- | --- | --- |
| Black | 16.7(15.7-17.6) | 18.2(17.3-19.1) | -0.06(-0.17-0.04) | 0.001 |
| White | 14.5(13.6-15.4) | 14.9(14.2-15.5) | -0.02(-0.13-0.08) | <0.001 |
| Mexican-American | 8.4(7.6-9.2) | 13.6(12.2-15) | -0.51(-0.61--0.40) | 0.032 |

**Appendix Table 10. Comparative Mean percentage of participants with asthma in** **hepatitis B surface antibody**

| **Variable** | **Hepatitis B surface antibody positive** | **Hepatitis B surface antibody negative** | **Mean Difference between asthma and non asthma** | **P Value** |
| --- | --- | --- | --- | --- |
| Black | 16.9(15.8-18) | 17.9(17.1-18.8) | -0.18(-0.07-0.28) | <0.001 |
| White | 16.5(15.4-17.6) | 14.2(13.6-14.8) | 0.06(-0.04-0.17) | 0.2 |
| Mexican-American | 11.3(10.2-12.5) | 9.1(8.3-9.8) | 0.11(0.08--0.22) | 0.009 |

**Appendix Table 11. Comparative Mean percentage of participants with asthma in** **hepatitis B core antibody**

| **Variable** | **Hepatitis B core antibody positive** | **Hepatitis B core antibody negative** | **Mean Difference between asthma and non asthma** | **P Value** |
| --- | --- | --- | --- | --- |
| Black | 12.5(10.7-14.2) | 18.1(17.3-18.9) | -0.28(-0.39--0.18) | 0.0016 |
| White | 14.3(11.3-17.3) | 14.9(14.4-15.5) | -0.10(-0.01-0.20) | 0.44 |
| Mexican-American | 5.4(3-7.8) | 9.9(9.2-10.7) | -0.40(-0.51--0.30) | 0.10 |

**Appendix Table 12. Comparative Mean percentage of participants with asthma in** **HSV-1 antibody**

| **Variable** | **HSV-1 antibody positive** | **HSV-1 antibody negative** | **Mean Difference between asthma and non asthma** | **P Value** |
| --- | --- | --- | --- | --- |
| Black | 16(14.6-17.4) | 19.5(17.7-21.3) | -0.16 (-0.26--0.05) | 0.048 |
| White | 15.9(14.7-17.2) | 16.8(15.9-17.7) | 0.01(-0.1-0.12) | 0.85 |
| Mexican-American | 7.7(6.7-8.6) | 13.8(11.8-15.8) | -0.49(-0.60--0.39) | <0.001 |

**Appendix Table 13. Comparative Mean percentage of participants with asthma in** **HSV-2 antibody**

| **Variable** | **HSV-2 antibody positive** | **HSV-2 antibody negative** | **Mean Difference between asthma and non asthma** | **P Value** |
| --- | --- | --- | --- | --- |
| Black | 17.3(15.7-19) | 16(14.5-17.5) | 0.17 (0.07-0.28) | 0.042 |
| White | 17.2(14.8-19.7) | 15.9(15-16.7) | 0.15(0.05-0.26) | 0.10 |
| Mexican-American | 8.1(6.3-10) | 8.2(7.1-9.3) | 0.13(0.02--0.24) | 0.43 |

**Appendix Table 14. Number and Proportion of Participants, by Distribution of** **Measles antibody and** **Rubella antibody.**

(a) Measles antibody

| Ethnic Group |  |  | Measles antibody | | |  |
| --- | --- | --- | --- | --- | --- | --- |
|  | <5 | 5-10 | 10-15 | 15-20 | 20-25 | >25 |
| Black, n(%) | 29.1(289) | 26.38(262) | 17.62(175) | 15.31(152) | 7.85(78) | 3.73(37) |
| White, n(%) | 51.97(580) | 22.94(256) | 14.96(167) | 7.35(82) | 1.79(20) | 0.99(11) |
| Mexican-American, n(%) | 47.02(276) | 25.72(151) | 16.01(94) | 8.18(48) | 2.9(17) | 0.17(1) |

(b) Rubella antibody

| Ethnic Group |  |  | Rubella antibody | | |  |
| --- | --- | --- | --- | --- | --- | --- |
|  | <50 | 50-100 | 100-150 | 150-200 | 200-250 | >300 |
| Black, n(%) | 56.9(565) | 29.1(289) | 11.48(114) | 1.91(19) | 0.5(5) | 0.1(1) |
| White, n(%) | 71.86(802) | 19.71(220) | 6(67) | 2.15(24) | 0.18(2) | 0.09(1) |
| Mexican-American, n(%) | 70.41(414) | 21.6(127) | 5.78(34) | 1.87(11) | 0(0) | 0.34(2) |

**Appendix Table 15. Baseline characteristics of participants.**

| variable | total | no | yes | Pvalue |
| --- | --- | --- | --- | --- |
| age | 37.15(0.18) | 37.48(0.19) | 35.23(0.30) | < 0.0001 |
| income | 2.85(0.03) | 2.87(0.03) | 2.70(0.04) | < 0.0001 |
| sex |  |  |  | < 0.0001 |
| Female | 52187(51.18) | 44569(50.59) | 7618(54.66) |  |
| Male | 50590(48.82) | 43438(49.41) | 7152(45.34) |  |
| eth |  |  |  | < 0.0001 |
| black | 24727(12.08) | 20247(11.63) | 4480(14.73) |  |
| mexican | 21580( 9.66) | 19510(10.22) | 2070( 6.36) |  |
| other | 18536(13.44) | 15771(13.39) | 2765(13.74) |  |
| white | 37934(64.82) | 32479(64.76) | 5455(65.16) |  |
| smoke |  |  |  | < 0.0001 |
| former | 14462(18.16) | 12432(24.27) | 2030(25.48) |  |
| never | 33060(40.24) | 28927(54.83) | 4133(50.06) |  |
| now | 12170(15.91) | 10215(20.91) | 1955(24.46) |  |
| education |  |  |  | < 0.001 |
| High school | 13619(17.68) | 11824(24.23) | 1795(22.93) |  |
| Less than high school | 15764(12.87) | 13914(17.76) | 1850(15.89) |  |
| More than high school | 29184(42.99) | 24944(58.01) | 4240(61.17) |  |

**Appendix Table 16. Associations between the vaccine and viral antibodies and asthma.**

| Character | Estimate | Std. Error | t value | Pr(>\|t\|) | OR | 95% CI |
| --- | --- | --- | --- | --- | --- | --- |
| Hepatitis A vaccine |  |  |  |  |  |  |
| yes | ref | ref | ref | ref | ref | ref |
| no | 0.124 | 0.042 | 2.935 | 0.004 | 1.132 | 1.132(1.041,1.231) |
| Hepatitis A vaccine 2 dose |  |  |  |  |  |  |
| yes | ref | ref | ref | ref | ref | ref |
| no | 0.251 | 0.106 | 2.372 | 0.019 | 1.285 | 1.285(1.043,1.583) |
| Hepatitis B vaccine |  |  |  |  |  |  |
| yes | ref | ref | ref | ref | ref | ref |
| no | 0.135 | 0.037 | 3.622 | <0.001 | 1.145 | 1.145(1.063,1.233) |
| Hepatitis B vaccine 3 dose |  |  |  |  |  |  |
| yes | ref | ref | ref | ref | ref | ref |
| no | 0.177 | 0.105 | 1.681 | 0.095 | 1.193 | 1.193(0.970,1.468) |
| Pneumonia vaccine |  |  |  |  |  |  |
| yes | ref | ref | ref | ref | ref | ref |
| no | 0.757 | 0.12 | 6.3 | <0.001 | 2.132 | 2.132(1.656,2.744) |
| HPV vaccine |  |  |  |  |  |  |
| yes | ref | ref | ref | ref | ref | ref |
| no | 0.297 | 0.087 | 3.416 | <0.001 | 1.346 | 1.346(1.132,1.600) |
| HPV vaccine dose |  |  |  |  |  |  |
| 1 | ref | ref | ref | ref | ref | ref |
| 2 | 0.078 | 0.201 | 0.387 | 0.714 | 1.085 | 1.085(0.724,1.626) |
| 3 | -0.104 | 0.177 | -0.592 | 0.566 | 0.916 | 0.916(0.648,1.278) |
| Hepatitis A antibody |  |  |  |  |  |  |
| yes | ref | ref | ref | ref | ref | ref |
| no | -0.146 | 0.041 | -3.563 | <0.001 | 0.864 | 0.864(0.797,0.937) |
| Hepatitis B surface antibody |  |  |  |  |  |  |
| yes | ref | ref | ref | ref | ref | ref |
| no | -0.221 | 0.3 | -0.735 | 0.463 | 0.802 | 0.802(0.443,1.451) |
| Hepatitis B core antibody |  |  |  |  |  |  |
| yes | ref | ref | ref | ref | ref | ref |
| no | -0.144 | 0.073 | -1.961 | 0.043 | 0.866 | 0.866(0.749,1.001) |
| HSV-1 antibody |  |  |  |  |  |  |
| yes | ref | ref | ref | ref | ref | ref |
| no | -0.11 | 0.051 | -2.165 | 0.032 | 0.895 | 0.895(0.809,0.991) |
| HSV-2 antibody |  |  |  |  |  |  |
| yes | ref | ref | ref | ref | ref | ref |
| no | 0.097 | 0.062 | 1.558 | 0.036 | 1.102 | 1.102(0.974,1.246) |
| Measles antibody | 0.018 | 0.006 | 2.819 | 0.007 | 1.018 | 1.018(1.005,1.032) |
| Rubella antibody | 0.001 | 0.001 | 1.451 | 0.047 | 1.001 | 1.001(1.000,1.003) |
| logistic regressiono analyses:adjusted for age,gender,eth,smoke,education and income. | | | | | | |
